# The Effects of Lipoic Acid on Yolk Nutrient Utilization, Energy Metabolism, and Redox Balance over Time in *Artemia* sp.

**DOI:** 10.3390/antiox12071439

**Published:** 2023-07-18

**Authors:** Juan Rafael Buitrago Ramírez, Robson Matheus Marreiro Gomes, Alan Carvalho de Sousa Araujo, Sonia Astrid Muñoz Buitrago, Jean Piraine Souza, José María Monserrat

**Affiliations:** 1 Programa de Pós Graduação em Aquicultura, Instituto de Oceanografia (IO), Universidade Federal do Rio Grande—FURG, Rua do Hotel, n° 2, Cassino, Rio Grande 96210-030, RS, Brazil; roobinho_matheus@furg.br (R.M.M.G.); alancsa@furg.br (A.C.d.S.A.); samunozb@unal.edu.co (S.A.M.B.); jeanpiraine@furg.br (J.P.S.); 2 Laboratório de Bioquímica Funcional de Organismos Aquáticos (BIFOA), Instituto de Oceanografia (IO), Universidade Federal do Rio Grande—FURG, Rua do Hotel, n° 2, Cassino, Rio Grande 96210-030, RS, Brazil; 3 Instituto of Ciências Biológicas (ICB), Universidade Federal do Rio Grande—FURG, Av. Itália, Km 08, Rio Grande 96201-900, RS, Brazil

**Keywords:** antioxidants, energy metabolism, nutritional supplements, nitrogen compounds

## Abstract

Lipoic acid (LA) is a mitochondrial coenzyme that, depending on the concentration and exposure time, can behave as an antioxidant or pro-oxidant agent and has a proven ability to modulate metabolism by promoting lipid and glucose oxidation for energy production. To assess the effects of LA on energy metabolism and redox balance over time, *Artemia* sp. nauplii was used as an animal model. The administered concentrations of the antioxidant were 0.05, 0.1, 0.5, 1.0, 5.0, and 10.0 µM. Therefore, possible differences in protein, triglyceride, glucose, and lactate concentrations in the artemia samples and total ammoniacal nitrogen (TAN) in the culture water were evaluated. We also measured the effects of LA on in vivo activity of the electron transport system (ETS), antioxidant capacity, and production of reactive oxygen species (ROS) at 6, 12, 18, and 24 h post-hatching. There was a decrease in glucose concentration in the LA-treated animals, and a decrease in ammonia production was observed in the 0.5 µM LA treatment. ETS activity was positively regulated by the addition of LA, with the most significant effects at concentrations of 5.0 and 10.0 µM at 12 and 24 h. For ETS activity, treatments with LA presented the highest values at 24 h, a period when ROS production decreased significantly, for the treatment with 10.0 µM. LA showed positive regulation of energy metabolism together with a decrease in ROS and TAN excretion.

## 1. Introduction

The manner in which animals transform matter and the energy provided in their diet varies widely depending on genotypic and environmental characteristics [1,2,3]. In general, animals in aquatic environments rely primarily on proteins as an energy source; thus, their utilization of protein synthesis and growth depends considerably on their energy requirements [4]. Consequently, the lipids and carbohydrates supplied by the diet play secondary roles as energy substrates. Thus, it is estimated that approximately 80% of the required ATP is synthesized by amino acid catabolism in fish [4,5]. Although lipids are important energetic substrates, they are primarily used during the preprandial period, especially when prolonged [6]. As for carbohydrates, there is a consensus that their contribution to ATP synthesis in aquatic organisms is low, although their use also increases during preprandial periods or prolonged fasting. Lipid mobilization is important for energy production [6,7], and this may be due to the low availability of digestible carbohydrates in natural aquatic environments [8]. There is a special interest in minimizing the protein used as an energy source in aquaculture because it comes from raw materials in the diet with a considerable economic cost, as it is a non-renewable ingredient extracted from the natural environment [9]. Thus, optimizing the use of amino acids from proteins for anabolic processes linked to development and growth through various strategies, such as supplementation with compounds capable of modulating metabolism, is an alternative approach to explore in the search for more sustainable aquaculture activities [10,11]. Supplemental feed within aquaculture is increasingly being used to improve the physiological parameters that influence production performance [12,13,14,15,16,17]. This requires greater importance to be placed on new trends, such as precision nutritional regulation under principles such as the optimization of feeding and cycling of generated organic matter to decrease pollutant emissions [18]. Precise nutritional regulation requires optimizing feed, products, and technical and technological support for waste and metabolic management [18]. Thus, nutritional supplements, which are understood as metabolic modulators, could become tools with which to couple physiology with the zootechnical demands of a species of productive interest [19,20,21]. There are compounds with the proven ability to significantly modulate the energy metabolism of aquatic species, including an increase in β-oxidation and glycolysis activity that can induce a protein-sparing effect [10,20,22]. Compounds with potential for this purpose include lipoic acid (LA), a well-known antioxidant with physiological benefits, as well as the ability to contain or decrease the impact of pro-oxidant events and modulate the metabolism of the body by prioritizing metabolic pathways for mitochondrial biogenesis, mobilization of energy substrates, and energy production [23,24]. However, lipoic acid undergoes a reductive reaction and produces dihydro lipoic acid, which consumes NADPH, thereby lowering the reductive potential in the cell. Thus, depending on dose and time, lipoic acid can also act as a pro-oxidant [25]. It has been noted that the mobilization of energy reserves caused by LA leads to a decrease in protein oxidation for energy production through the positive regulation of glycolysis and β-oxidation [11,26,27]. In aquaculture, LA can be used as a promoter of protein efficiency and as a potential supplement with which to decrease the protein requirements of cultured organisms [11,26]. Although there is evidence that LA promotes protein efficiency in aquatic and terrestrial animals, the results of Terjesen et al. (2004) [28] diverge from this idea, which may point to the possible interactions of LA with the metabolism of the species and diet provided [22,29,30]. However, the possible effects of a diet that is not adjusted to the animal’s requirements could lead to inconclusive results regarding how LA functions at the metabolic level. Thus, a strategy is needed at the experimental level to avoid possible misunderstandings in the assessment of the metabolic effects of LA, such as using animal models at stages where they depend on yolk reserves and adjusting to the requirements of animals at the predetermined stage of development [31,32]. Therefore, this study aimed to evaluate the metabolic changes in the use of nutrients in newly hatched *Artemia* sp. nauplii treated with LA and the effect of this compound on the redox balance of the animals. The choice of artemia nauplii as an animal model is due to the fact that this animal depends on yolk reserves, meaning that there is a supply of LA within a nutritional context where the species’ requirements are met.

## 2. Materials and Methods

### 2.1. Artemia sp.

Artemia cysts were incubated in Imhoff cones to hatch at a ratio of 1 g L^−1^ cysts at 28 °C, 28 g L^−1^ salinity, aeration, and constant light. After 24 h of incubation, the newly hatched nauplii were collected in a single beaker, from which two aliquots of 1 mL were diluted in 99 mL seawater. From these diluted suspensions, 1 mL was taken with a glass pipette and the number of organisms was counted in duplicate. The average counts from each 100 mL beaker were calculated. The result was multiplied by 100 to determine the number of artemia mL^−1^ in the beaker. Thus, the density of artemia was adjusted to 250 mL^−1^ by replacing 100% of the water in the collection beaker with water treated with 15% sodium hypochlorite (final concentration 0.015%) and dechlorinated with sodium thiosulfate.

### 2.2. Standardization of the In Vivo Electron Transport System (ETS) Activity Protocol

This protocol was partially based on the protocol described by [33] Reid et al. (2018) using *Danio rerio*. This procedure uses resazurin as a fluorophore, which, when reduced by ETS mitochondrial proteins, is transformed into resorufin, a fluorescent substance (excitation: 530 nm; emission: 590 nm). The variation in fluorescence per minute was considered an indicator of the ETS activity. To standardize the artemia protocol, the influence of the resazurin concentration (0.5, 0.75, and 1.00 mg mL^−1^ final concentrations) and animal density (60, 120, and 240 artemia nauplii mL^−1^) on fluorescence readings was tested using a full factorial design with three replicates per treatment. Readings were performed on white 96-well plates at 28 °C for 1 hour with a reading frequency of 2 min using a Synergy HT spectrofluorimeter (BioTek, São Paulo, Brazil). To assess the sensitivity of the protocol to changes in ETS activity, the effect of potassium cyanide (KCN), an inhibitor of ETS complex IV, on fluorescence kinetics was tested. Thus, final KCN concentrations of 50, 100, 150, and 200 µM were used. Immediately after KCN was added to the nauplii arranged in 96 white plate wells (240 nauplii mL^−1^), resazurin (0.01 mg mL^−1^) was added to nine wells of each KCN concentration and nine wells that received only distilled water as the KCN vehicle. Additionally, dichlorofluorescein diacetate (H_2_DCF-DA) was used at a final concentration of 8.3 µM [34] and read at 485 nm (excitation) and 520 nm (emission) in nine additional wells exposed to different KCN concentrations or distilled water to verify ROS production as an additional marker of mitochondrial activity to confirm the effect of KCN on mitochondria using a standardized method. It is worth emphasizing that the nauplii remained alive after ETS and ROS measurements, including those exposed to KCN.

#### 2.2.1. Isolation of the Mitochondrial Fraction

Mitochondria were extracted from artemia to estimate their capacity to reduce resazurin in vivo. This analysis was designed to quantitatively determine the contribution of the mitochondrial fraction of artemia to in vivo ETS activity. Mitochondrial fractions were isolated in triplicate from three samples of artemia incubated separately under the same conditions as those previously reported. Additionally, non-mitochondrial fractions were recovered to evaluate their contribution to the fluorescence observed in the live artemia specimens.

Two g of newly hatched artemia nauplii were gently homogenized using a Teflon homogenizer in 16 mL of buffer 1 (0.125 M sucrose, 0.375 M sorbitol, 1 mM EGTA, 150 mM KCl, 0.5% bovine serum albumin free of fatty acids, and 20 mM HEPES KOH, pH 7.5) which had been cooled with ice [35]. Subsequently, the homogenate was centrifuged for 10 s at 3026× *g* and 4 °C. The supernatant was recovered and centrifuged for 15 min at 17,409× *g* and 4 °C. After discarding the supernatant from the previous centrifugation, the pellet was resuspended in 32 mL of buffer 2 (0.125 M sucrose, 0.375 M sorbitol, 0.025 mM EGTA, 150 mM KCl, 0.5% acid-free bovine serum albumin fatty acids, and 20 mM HEPES KOH, pH 7.5) and centrifuged at 1082× *g* and 4 °C for 5 min. After centrifugation, the supernatant was recovered and centrifuged for 15 min at 17,409× *g* and 4 °C. The generated pellet (mitochondrial fraction) was resuspended in 300 µL of buffer 2 [35].

#### 2.2.2. Measurement of the Reductive Capacity of Artemia Mitochondria

From an aliquot of the mitochondrial fraction, the protein concentration of the mitochondrial fraction was determined using the Biuret method. Subsequently, in white 96-well plates, the reaction medium (5 mM sodium succinate; 0.125 M sucrose; 0.065 M KCl; 0.002 M K_2_HPO_4_; and 0.01 M KOH-HEPES, pH 7.5) plus distilled water, adenosine diphosphate (ADP; final concentration: 103 µM), or ADP 103 µM + KCN as inhibitor (final concentration: 103 µM) along with resazurin (final concentration: 32.5 µM) were added in sequence. Finally, buffer 2 was added to measure the blank for the analysis: fraction 1 (pellet resulting from the first centrifugation), fraction 2 (fractions discarded from subsequent centrifugations), and mitochondrial fraction, all of which had a final concentration of 1 mg of protein mL^−1^. The generated fluorescence was used to measure the reducing mitochondrial capacity of resazurin (530 nm excitation and 590 nm emission) for 10 min at 28 °C, with readings taken every 1 min [35,36]. The fluorescence data were multiplied by the amount of protein in each fraction, as follows:

mT_mit_ = m_mit_ × prot_mit_·g^−1^ of brine shrimp

mT_f1_ = m_f1_ × prot_f1_·g^−1^ of brine shrimp

mT_f2_ = m_f2_ × prot_f2_·g^−1^ of brine shrimp

where mT_mit_, mT_f1_, and mT_f2_ represent the slopes of the mitochondrial fraction, fraction 1, and fraction 2, respectively, for the total amount of protein present in each fraction for 1 g of brine shrimp. In turn, m_mit_, m_f1_, and m_f2_ represent the slopes of the fluorescence units obtained for the mitochondrial fraction, fraction 1, and fraction 2 exposed to succinate + ADP, respectively. Multiplying the values of m_mit_, m_f1_, and m_f2_ by the amount of total protein in each fraction present in 1 g of brine shrimp (prot_mit_·g^−1^ of *Artemia* sp. nauplii, prot_f1_·g^−1^ of *Artemia* sp. nauplii, and prot_f2_·g^−1^ of *Artemia* sp. nauplii, respectively), the values of mT_mit_, mT_f1_, and mT_f2_ were estimated. Based on the mT_mit_, mT_f1_, and mT_f2_ values, the percentage of participation of each fraction in the resazurin reduction rate was calculated. Additionally, statistical differences between the slopes of the fluorescence lines for each substrate or inhibitor (succinate, succinate + ADP, and succinate + ADP + KCN) for each fraction were evaluated.

### 2.3. In Vivo Exposure of Artemia to Lipoic Acid (LA)

Two experiments were performed to evaluate the effects of LA on artemia nauplii. In the first experiment, we exposed the animals to LA to assess changes in nutrient reserve consumption in the yolk, antioxidant capacity, and ETS for 24 h post-hatching. This duration was selected because, according to [37] (1967), 30 h after hatching, artemia salina had nearly consumed their yolk and obtained their first food. In the second experiment, artemia nauplii were exposed to LA, and the ETS and ROS concentrations were measured after 18 and 24 h. This is because, in animals at 12 h or less post-hatching, the ETS kinetic readings showed determination coefficients below 40% (see Section 4).

#### 2.3.1. Experiment 1

In 8 24-well plates, 2 mL of artemia nauplii was stocked (24 h post-rehydration) per well at a density of 250 artemia nauplii mL^−1^, and 6 plates were treated with final concentrations of 0.05, 0.1, 0.5, 1.0, 5.0, and 10.0 µM. One of the plates was used as a control for the LA vehicle, which was treated only with dimethyl sulfoxide (DMSO) at a final concentration of 0.005%. The remaining plate, to which only distilled water was added, was used as the experimental control. Nauplii were collected from three wells of each treatment every six hours for 24 h, filtered with 60 µm pore size screens, deposited into previously weighed 2 mL microtubes, quickly submerged in liquid nitrogen, and stored at −80 °C for further analysis. The water from each well was used to measure the total ammonia nitrogen (TAN) concentration by means of the phenol-hypochlorite method [38]. A calibration curve with ammonium chloride obtained after the analysis of the water samples was used to quantify the TAN. Owing to interference from DMSO in the reaction, DMSO was added to the standard curve at the concentration used for the experiment.

#### 2.3.2. Experiment 2

Artemia nauplii were stocked in 4 96-well plates 24 h post-rehydration at 240 organisms mL^−1^. Twelve wells per plate were exposed to 0.05, 0.1, 0.5, 1.0, 5.0, and 10.0 µM lipoic acid or the LA vehicle dimethyl sulfoxide (DMSO) at a final concentration of 0.005%. In each case, only 20 µL of each solution was added to the wells to avoid extreme dilution of the water salinity (the dilution factor was 1%). Because the metabolic rate of artemia nauplii remained low at 6 and 12 h, ETS activity and ROS generation were only evaluated at 18 and 24 h. Thus, the fluorophores resazurin and H_2_DCF-DA were added to the plates, with six wells for each fluorophore and each LA concentration or control. The fluorescence of each fluorophore was read every 2 min at the previously mentioned lengths for 3 h to obtain the fluorescence variations per minute and per well, following the study of Rodrigues et al. (2021) [34].

### 2.4. Biochemical Analysis

#### 2.4.1. Sample Processing

The microtubes with artemia nauplii were weighed, and the microtubes’ weights were recorded to determine the weights of the collected samples. The samples were diluted five times with buffer (0.09 M Na_2_HPO_4_, 0.09 M KHPO_4_, 0.45 mg mL^−1^ polyvinylpyrrolidone, 22.5 µM MgSO_4_, and 0.16% Triton X-100) and sonicated at 40 kHz with a 3 mm diameter tip in 30 s pulses for 3 min while kept permanently on ice. After homogenization, samples were centrifuged at 2500× *g* for 10 min at 4 °C. The supernatants were stored in 500 µL microtubes at 80 °C for further analysis.

#### 2.4.2. Determination of Protein Concentration

For protein analysis, the Bioclin kit for total protein was used. In 1.5 mL microtubes, 7.5 µL of sample supernatant, the homogenization buffer (blank), or a solution of 40 mg of albumin mL^−1^ (protein standard), as well as 375 µL of Biuret reagent, was added [39]. The resulting solutions were then homogenized using a vortex and incubated for ten minutes before being transferred to 96 transparent flat-bottom microplates using two wells per sample, including blank and standard. The absorbance was measured at 550 nm using a spectrofluorometer, and the protein concentration of the samples was calculated based on the absorbance obtained from the standard protein solution. The protein results are expressed in mg g^−1^ of artemia nauplii.

#### 2.4.3. Determination of Glucose Concentration

The dosage was determined using the Bioclin Monoreagent kit (Porto Alegre, RS, Brazil) according to the manufacturer’s instructions. Homogenates of artemia nauplii samples, homogenization buffer as an analysis blank, and a standard glucose solution at 1 mg mL^−1^ were used. The procedure was performed in 96-well transparent plates, and after adding the monoreagent for the glucose samples and aliquots in duplicate, the plates were incubated at 37 °C for 10 min and read at 505 nm. The glucose concentration in the samples was calculated based on the absorbance of the standard glucose solution. The glucose results are expressed in mg g^−1^ of artemia.

#### 2.4.4. Determination of Lactate Concentration

This was aided by a commercial kit for lactate (Bioclin, Porto Alegre, RS, Brazil) based on the production of NADH after the lactate and the NAD^+^ reaction to generate pyruvate + NADH, catalyzed by the enzyme lactate dehydrogenase. NADH production was measured fluorometrically for each sample in duplicate at 340 and 440 nm. A standard lactate solution (0.3 mg mL^−1^) was used, from which six serial dilutions were made up to a concentration of 0.004 mg mL^−1^, with which a calibration curve was constructed. The lactate concentrations of the samples were measured five minutes after the addition of the lactate dehydrogenase enzyme and NAD^+^ solution, and were then calculated against the fluorescence obtained from the lactate calibration curve. Readings were taken on 96-well white plates and each sample was replicated three times. The lactate results are expressed in mg g^−1^ of artemia nauplii.

#### 2.4.5. Determination of Triglyceride Concentration

The analysis was performed with the initial undiluted artemia nauplii homogenates using a commercial monoreagent kit from Bioclin (Porto Alegre, RS, Brazil). The analysis was performed in 96-well transparent plates. The samples, blank, and triglyceride standard (1 mg mL^−1^) were analyzed in duplicate. Next, the plates were incubated at 37 °C for five minutes, and the absorbance was measured at 500 nm. The triglyceride concentrations in the samples were calculated based on the absorbance of the standard triglyceride solution. The results are expressed as artemia mg triglycerides g^−1^.

#### 2.4.6. Determination of Total Antioxidant Capacity

The analysis of antioxidant capacity against peroxyl radicals was performed with samples adjusted to a concentration of 0.5 mg of protein mL^−1^. The results are expressed as the relative area of the fluorescence curves generated over time by the oxidation of H_2_DCF-DA (Sigma-Aldrich, St. Louis, MO, USA) in the presence of the sample with or without the peroxyl radical generator, 2,2-azobis-2-methylpropionamidine dihydrochloride (ABAP Sigma-Aldrich). In this analysis, the larger the relative area obtained, the lower the antioxidant capacity of the sample, and vice versa [40].

### 2.5. Statistical Analysis

After checking the assumptions of normality, homoscedasticity, and independence of the variables, two-way analysis of variance (ANOVA) was performed (time and LA concentration) and Bonferroni’s post hoc test was used to compare the means of each treatment. For the experimental control without a vehicle, a single mean was calculated for each evaluated response variable, grouping all collection times. Therefore, these data were not included in the comparisons between the treatments. The slopes of the reductive mitochondrial capacity isolated from artemia nauplii experiments were compared using ANOVA and Tukey’s contrasts after verification of normality and variance homogeneity (using the Shapiro–Wilk and Levene tests, respectively). Finally, principal component analysis (PCA) was performed with the mean of each experimental group for each time, scaling the variables used with a mean of 0 and a standard deviation of 1. In the statistical tests, a significance level of 0.05 was adopted. In this sense, results with *p*-values lower than 0.05 were considered significant.

## 3. Results

### 3.1. Standardization of Activity Protocol for the In Vivo Electron Transport System (ETS)

**Figure 1:**
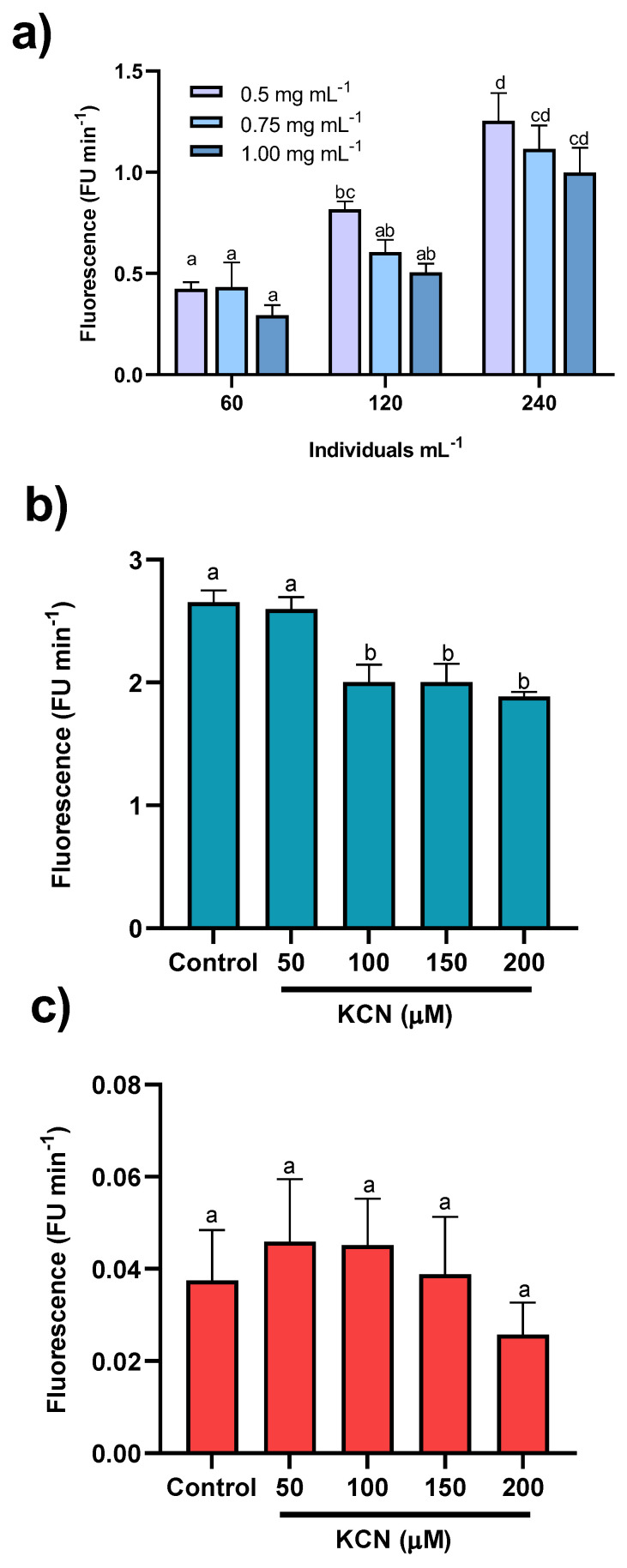
(**a**) Fluorescence increment per minute using different densities of artemia nauplii and different resazurin concentrations. Data are expressed as mean ± standard error (n = 3). (**b**) Effect of KCN on the electron transport system (ETS) activity. (**c**) Effect of KCN in reactive oxygen species (ROS) production. In (**b**,**c**), data are expressed as the mean ± standard error (n = 9). In all cases, different letters indicate statistically significant differences (*p* < 0.05) between the treatments.

Figure 1a shows the significant effect of animal density on the increase in fluorescence units per minute. However, the resazurin concentration did not have a significant effect (*p* > 0.05). KCN exposure, as seen in Figure 1b (*p* < 0.050), promoted a dependent response in terms of ETS determination using resazurin, whereas for ROS production, there were no differences between KCN concentrations (Figure 1c; *p* > 0.05).

### 3.2. Measurement of the Reductive Capacity of Artemia Nauplii Mitochondria

**Figure 2:**
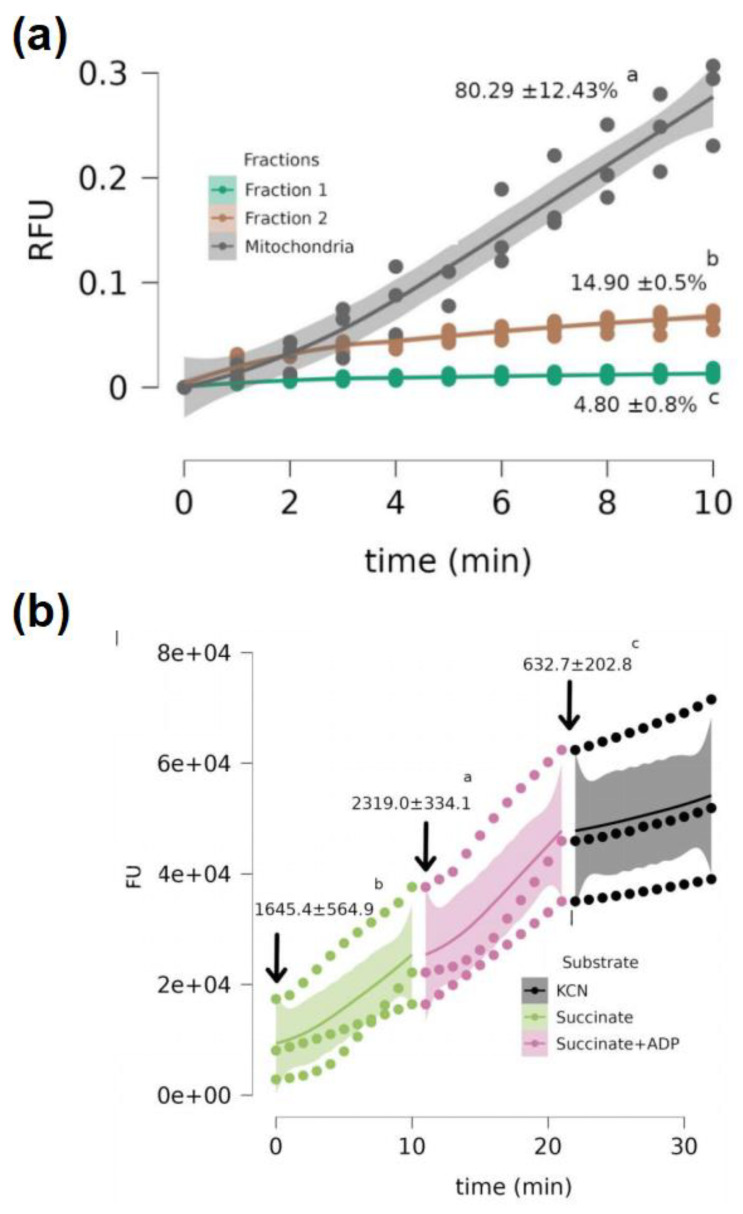
(**a**) Slopes of fluorescence readings (RFU) over time (min) estimated for fractions 1, 2, and the mitochondrial fraction exposed to succinate + ADP. The percentage values ± SD indicate the contribution of each fraction to the net fluorescence increment per min and per mg of protein contained in 1 g of artemia nauplii. Different letters indicate differences between the slopes of different fractions (*p* < 0.05). (**b**) Response of the mitochondrial fraction to different substrates. Values ± SD indicate the mean slopes of fluorescence increment per mg of protein contained in 1 g of artemia nauplii for each evaluated substrate. Colored bands represent the 95% confidence intervals calculated for each substrate. Different letters represent significant differences between the means of the slopes (*p* < 0.05).

All fractions reduced resazurin. However, the reduction rates found in the mitochondrial fraction were, on average, approximately five times higher than those found in the other fractions. Percentage-wise, the mitochondrial fraction exposed to succinate and ADP represented 80.29 ± 12.43% of the total resazurin reduction slope, followed by fraction 2 (14.90 ± 0.5%) and fraction 1 (4.80 ± 0.8%) (Figure 2a). For the mitochondrial fraction, significant differences were observed between the fluorescence slopes per minute, depending on the type of substrate or inhibitor used. The mitochondrial fraction exposed to succinate + ADP showed the highest resazurin reduction rate, followed by that exposed only to succinate. The samples exposed to succinate + ADP + KCN showed the lowest fluorescence growth rates (Figure 2b).

### 3.3. Experiment 1

#### 3.3.1. Protein Concentration

**Figure 3:**
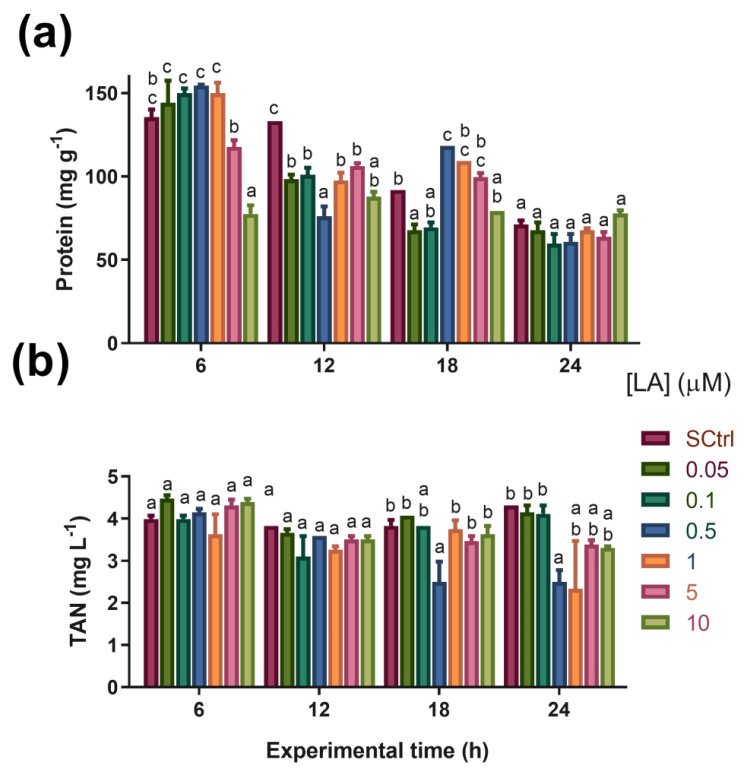
(**a**) Protein levels in artemia nauplii at different experimental times (h). (**b**) Total ammoniacal nitrogen (TAN) concentration in the water over time (h). Data are expressed as mean ± standard error (n = 3). Different letters indicate statistically significant differences (*p* < 0.05) between treatments. SCtrl: solvent control. Bars of different colors indicate exposure to different concentrations of lipoic acid (μM).

The results are shown in Figure 3a. At six hours, only the 10 µM treatment showed a lower protein concentration than the SCtrl treatment (control with DMSO) and the other LA treatments. At 12 h, all groups treated with LA showed lower protein levels than the SCtrl group. However, at 18 h, the treatment with 0.5 µM LA showed higher protein levels than SCtrl, which contrasted with the values obtained by the treatment with 0.05 µM, which were lower than those of SCtrl. After 24 h, no differences were found between the treatments.

#### 3.3.2. Total Ammoniacal Nitrogen (TAN)

At 18 and 24 h, there were decreases in the TAN concentration in the water of the treatment with 0.5 µM LA compared to SCtrl (Figure 3b).

#### 3.3.3. Glucose Concentration

**Figure 4:**
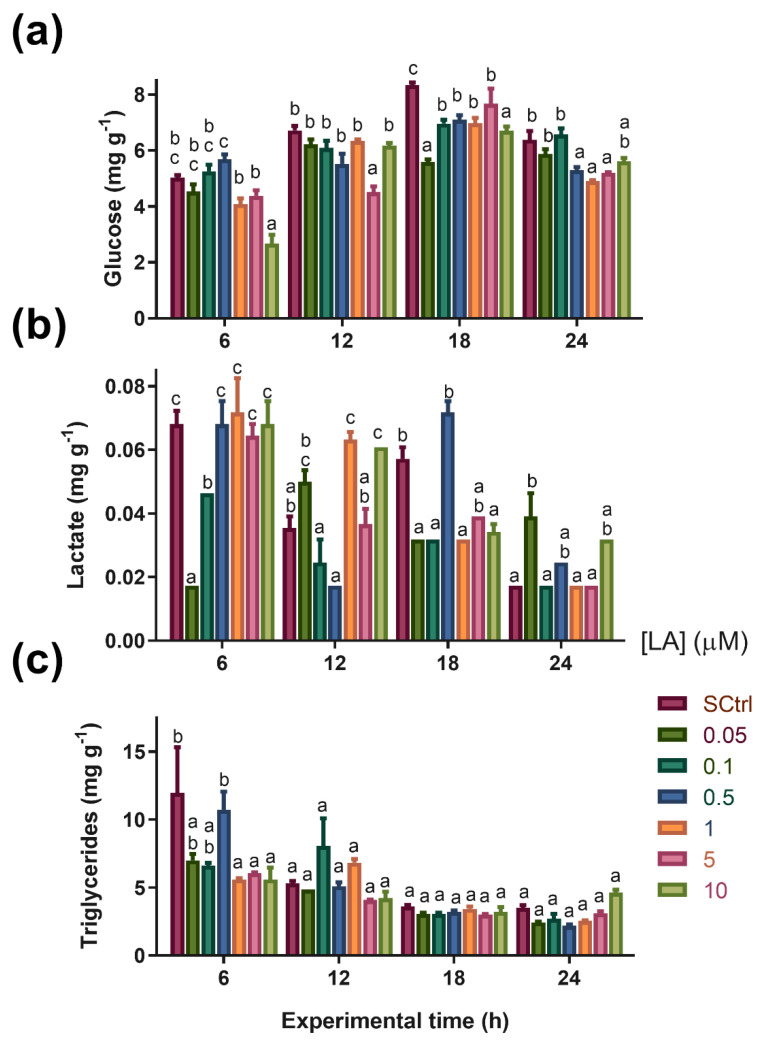
Glucose (**a**), lactate (**b**), and triglyceride (**c**) levels over time (h) in artemia nauplii. Data are expressed as means ± standard error (n = 3). Different letters indicate statistical differences (*p* < 0.05) between treatments. SCtrl: solvent control. Bars of different colors indicate exposure to different lipoic acid concentrations (in μM).

The results for glucose are shown in Figure 4a. For the six-hour duration, the treatment with 10 µM showed significantly lower glucose values than in SCtrl; therefore, for the twelve-hour duration, the treatment with 5 µM LA demonstrated the lowest glucose values. At 18 h, all groups that received LA showed lower glucose values than those found in SCtrl, and at 24 h, only treatments with 0.5, 1, and 5 µM LA showed differences compared to SCtrl.

#### 3.3.4. Lactate Concentration

The lactate concentration showed differences between treatments. The treatments conducted for six hours presented the lowest lactate values, with 0.05 and 0.1 µM LA. At 12 h, there was an increase in lactate in the 1 and 10 µM treatments compared to that in SCtrl. At 18 h, the lactate values of the treatments with LA were generally lower than those presented by SCtrl, except for the treatments with 0.5 and 5 µM LA. Finally, at 24 h, the 0.05 µM LA treatment showed lactate values above those presented by SCtrl (Figure 4b).

#### 3.3.5. Triglyceride Content

The triglyceride content decreased at six hours in the groups treated with the highest LA concentrations (1, 5, and 10 µM LA) (Figure 4c).

#### 3.3.6. Total Antioxidant Capacity

**Figure 5:**
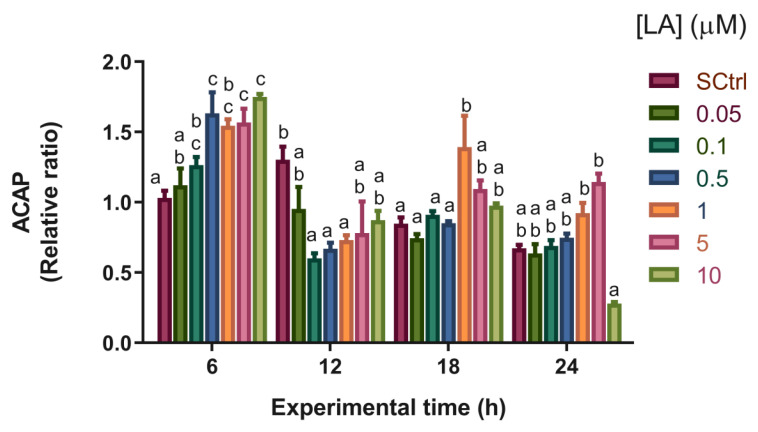
Total antioxidant capacity against peroxyl radicals (ACAP) in artemia nauplii over time (h). Data are expressed as means ± standard error (n = 3). Different letters indicate statistical differences (*p* < 0.05) between treatments. SCtrl: solvent control. Bars of different colors indicate exposure to different lipoic acid concentrations (in μM).

Overall, LA decreased the antioxidant capacity (ACAP). An increase in the antioxidant capacity was observed for the 10 µM treatment at the 24 h time point compared to the 1 and 5 µM treatments (Figure 5).

#### 3.3.7. PCA Analysis

**Figure 6:**
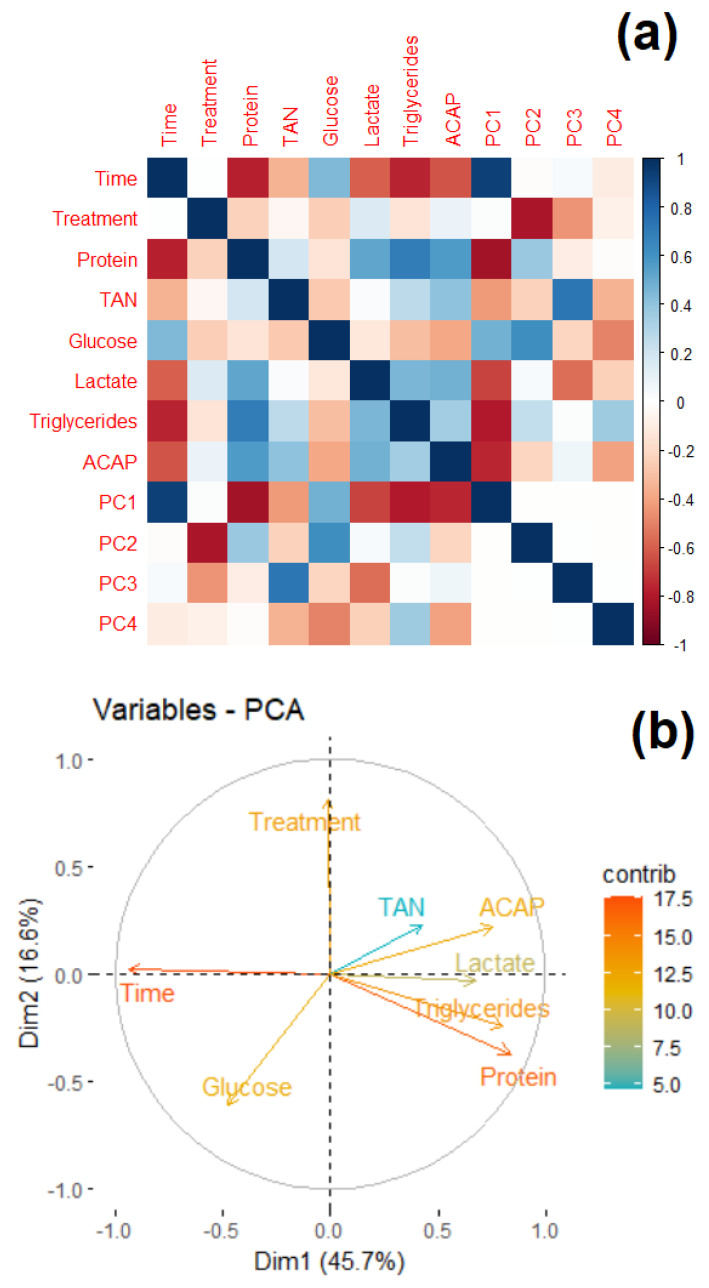
PCA results. (**a**) The correlation matrix is transformed into a color scale. (**b**) Contribution of each variable to the percentage of variance explained by the main components PC1 (x-axis) and PC2 (y-axis).

The first four PCA components explained 84.95% of the variance in the data matrix (PC1 45.65%, PC2 16.64%, PC3 13.69%, and PC4 8.95%). Positive correlations were found between PC1 and time (94%), as well as glucose (48%), and negative correlations were observed for protein (−87%), TAN (−43%), lactate (67%), triglycerides (−80%), and ACAP (76%). For PC2, two variables were strongly and negatively correlated with LA concentration (−81%) and positively correlated with glucose (61%). PC3 showed the highest correlation with TAN (73%), glucose, and lactate (−56%). PC4 showed the highest correlation with glucose (−49%) and ACAP (−40%). Protein levels correlated positively with lactate and triglycerides (53 and 70%, respectively) and negatively with time (77%). Triglycerides were negatively correlated with time (−76%) and glucose (−31%), and positively correlated with lactate (45%). ACAP was negatively correlated with time (−62%) and positively correlated with protein (57%), TAN (40%), lactate (48%), and triglycerides (34%) (Figure 6).

### 3.4. Experiment 2

**Figure 7:**
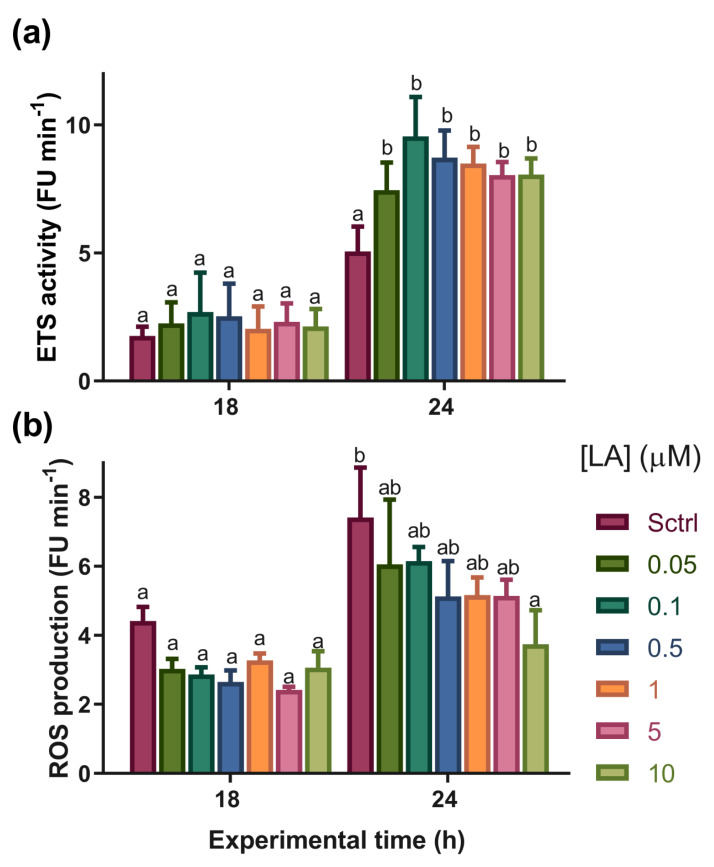
(**a**) Electron transport system activity and (**b**) reactive oxygen species (ROS) concentration at 18 and 24 h in artemia nauplii. Data are expressed as means ± standard error (n = 3). Different letters indicate statistical differences (*p* < 0.05) between treatments. SCtrl: solvent control. Bars of different colors indicate exposure to different lipoic acid concentrations (in μM).

The LA concentration and experimental time influenced the parameters of ETS activity and ROS production, and a significant interaction (*p* < 0.05) was observed between the LA treatments and exposure time for the variable ETS activity. Regarding this variable, at 18 h, no differences were detected (*p* > 0.05) among the treatments, whereas at 24 h, all treatments with LA showed higher ETS activity (*p* < 0.05) than SCtrl, with no differences among them. For ROS production at 18 h, it was not possible to observe differences between the experimental groups; however, after 24 h, there was a decrease in ROS production in the treatment with 10 µM compared to SCtrl (*p* < 0.05) (Figure 7).

## 4. Discussion

The standardization results of the resazurin protocol for kinetic analysis of ETS activity indicate that this protocol can be used to evaluate the ETS function of artemia nauplii. In animals 12 h or less post-hatching, the kinetic readings showed determination coefficients below 40%. Therefore, we do not recommend using the protocol during this period of life, as this phase should be associated with a low metabolic rate of the animals. At 16 h post-hatching, readings with determination coefficients above 90% were observed, which was the time taken to perform the ETS measurements. As expected, the density of the animals had a significant effect on the kinetics, indicating that the appearance of fluorescence is dependent on the metabolic activity of the animals contained within the well. However, the resazurin concentrations tested did not significantly affect the fluorescence generated when a final concentration of 0.005 mg mL^−1^ was used, which was four times lower than the concentration used by Reid et al. (2018) [33] for *Danio rerio* fish. This concentration did not produce toxic effects in either the larval or adult stage. Thus, it can be assumed that resazurin concentration allows for reliable estimation of the organism’s metabolism without introducing technical artifacts. The lack of an effect of KCN on ROS levels (Figure 1c) could be associated with the existence of alternative oxidases (AOX) [35], as discussed below. The analysis performed after the isolation of the mitochondrial fraction of artemia nauplii showed that the highest net reduction of resazurin occurred in the mitochondrial fraction. This result indicates the feasibility of the method of quantifying in vivo mitochondrial activity in artemia nauplii, at least for the first hours of life, as previously described in *D. rerio* [33]. Regarding the mitochondrial fraction, the results indicated significant effects of the substrates on the resazurin reduction. By comparing the obtained results with those generally expected from mitochondrial respiratory activity, it is possible to explain the effects of each substrate on resazurin reduction. In the case of succinate, an ETS substrate that enters complex II to be oxidized as an electron donor increases the respiratory activity of the mitochondria. When this occurs in the absence of ADP, the rates of oxygen consumption, despite existing, are low (stage 4 of mitochondrial respiration), which leads to reduced mitochondrial capacity (Figure 2b). However, with the addition of ADP (stage 3 of mitochondrial respiration), an increase in oxygen consumption was expected to lead to an increase in the reducing capacity of mitochondria (Figure 2b). Thus, the obtained results were consistent with the bioenergetic mechanics of mitochondria, suggesting that resazurin is a reliable tool for measuring in vivo mitochondrial activity. Using KCN, the reductive mitochondrial capacity was not completely abolished. Rodriguez-Armenta et al. (2018) mentioned that the use of cyanide and octyl-gallate were necessary to induce a full inhibition of *A. salina* mitochondrial oxygen consumption, suggesting that an alternative oxidase may be present in this organism. In this study, the authors reported that when KCN was added first, it partially inhibited mitochondrial oxygen consumption (in our case, mitochondrial reductive capacity, Figure 2b), and was completely inhibited after adding octyl-gallate. As mentioned previously [41,42], oxyconformers present branched mitochondrial respiratory electron chains. Together with the well-known mitochondrial oxidative phosphorylation (OxPhos) components, other redox enzymes are present as alternative oxidases (AOX), which, as mentioned above, are inhibited by other molecules such as octyl-gallate [35]. It is also important to note that Talbot et al. (2008) [42] mentioned that cellular sites where resazurin is reduced include the mitochondrial matrix. A study by Springer et al. (1998) [43] showed that the inhibition of resazurin reduction occurs either by inhibiting complex I (using antimycin A) or complex II (using malonate). It is known that the reduction potential (E_0_ at pH = 7.0 and temperature = 25 °C) of resazurin is +380 mV, which is reduced by molecules such as NAD(P)H (E_0_ = +320 mV), FADH_2_ (E_0_ = +220 mV), and cytochromes (E_0_ ranging from −80 to +290 mV) [44]. It should be noted that cyanide, as well as other poisons, like CO, arrest the whole process of mitochondrial respiration, reducing the oxidation of NADH or FADH_2_ and, thus, decreasing the electron flow to reduce resazurin (Figure 2b). Regarding the protein values observed in Experiment 1, the greatest changes were observed over the exposure time, presenting a gradual decrease in their values. LA influenced the values of this parameter, although to a lesser extent than time, which can be explained by the fact that the animals were fasting. In this situation, the available protein is likely to be used as an energy source and reduced to amino acids for the resynthesis of protein or for the production of osmolytes such as taurine, which is synthesized from methionine [45,46,47]. Given that the protein concentration patterns of the LA-treated groups changed at different exposure times compared with SCtrl (Figure 3a), it is possible to consider the interactions between the developmental stage of artemia and LA as a metabolic modulator. Most of the mass contained in yolk platelets is in the form of proteins [48], which need to be degraded into amino acids that serve as the forming units of new proteins for nauplii [48]. This process is catalyzed in the nauplii phase, primarily by thiol proteases, such as cathepsin B-like [49]. There is evidence of redox regulation by cathepsin B because molecules with thiol groups, such as reduced glutathione (GSH), can elevate proteolytic activity in purified bovine cathepsin B [49]. In the case of reduced LA (dihydrolipoic acid or DHLA), concentrations between 1 and 10 µM have been observed to elevate proteolytic activity by up to 80% of the maximum enzyme activity in a solution with 2 mM GSH [49]. The induced effect of LA on yolk protein degradation could subsequently favor the protein degradation of yolk platelets for protein synthesis in the nauplii, as was observed at 18 h in the group supplemented with 0.5 µM (Figure 3a).

Regarding TAN concentration in water, the decrease observed in the treatment with 0.5 µM LA after 18 and 24 h may indicate a decrease in the deamination of amino acids for use as an energy source, possibly allowing greater efficiency to be achieved in protein utilization for artemia development. This hypothesis could explain why the concentration of 0.5 µM LA showed significantly lower TAN concentrations in water and higher protein concentrations at the 18 h time point. LA has been observed to negatively regulate the expression of proteins related to oxidative amino acid metabolism in experiments performed with other species. In carp *Ctenopharyngodon idellus*, decreased expression of the enzymes aspartate aminotransferase and alanine aminotransferase was observed when supplemented with 600 and 1200 mg LA Kg^−1^ in the diet [11]. Both proteins actively participate in amino acid catabolism and can be used as markers of the intensity of this process [11,50]. In aquaculture, lower rates of protein utilization as an energy substrate promote mass protein gain in animals, and can have considerable repercussions in terms of decreasing the protein requirement of the diet [9]. In addition, lower ammonia production influences the reduction in effluents rich in nitrogenous compounds from aquaculture and, thereby, the sustainability of the activity [51]. However, a disclaimer observation must be stated: no differences in protein levels at 24 h were observed between treatments (Figure 3a), even when the TAN level of the group exposed to 0.5 µM LA was lower than that of the control group (Figure 3b), a result that requires further confirmation.

In the case of triglycerides, it was not possible to observe a considerable effect of LA compared to SCtrl over the experimental period. Throughout the experiment, there was only one peak with a higher triglyceride concentration in the treatment with 0.5 µM LA in the first six hours (Figure 4c). In artemia, most lipid fractions are stored as neutral lipids (80%), with triglycerides representing approximately 70% [52]. Triglyceride utilization is mainly divided into the synthesis of structural lipids and the supply of free fatty acid for energy production [45]. Although these results do not show a significant effect of LA on triglyceride concentration, unlike other existing studies [11,53], similar responses to those found herein have been observed in other aquatic animal species, including crustaceans. For example, in the crab *Eriocheris sinensis*, two studies have reported that LA has no significant effect on the lipid content in this species [27,54].

In the case of glucose, there was an overall gradual increase in glucose levels until the 18 h time period, and from the 24 h time period (Figure 4a), there was a general decrease in the levels of this energy substrate. This behavior can be explained by the amount of trehalose in artemia cysts. Trehalose is a disaccharide composed of glucose monomers and is stored at high concentrations inside cysts, acting as a cellular protector during the cryptobiosis phase [55]. However, when the organism returns to development after rehydration, trehalose is hydrolyzed by the trehalase enzyme and transformed into glucose, which acts as an energy substrate to supply accelerated metabolic activity during development [56,57]. It is worth noting that glucose showed a negative correlation with the protein and triglycerides variables according to the results obtained by PCA (Figure 6), since, while the levels of protein and triglycerides decreased, glucose concentrations increased at least until the 18 h time point, perhaps due to increased gluconeogenesis. This could indicate that proteins and triglycerides participate as substrates for energy metabolism under normal physiological conditions. From this moment on, glucose has begun to occupy a place of greater importance as an energy substrate. In addition, it is particularly interesting to note that during the experiment, there were always groups treated with LA with glucose concentrations lower than those presented by the SCtrl group, corroborating the importance of LA as a promoter of carbohydrate metabolism [22,24]. These results seem to agree with those obtained for *Macrobrachium nipponense*, in which LA supplementation (700 and 1400 mg Kg^−1^ feed) was found to increase the expression of glycolysis, Krebs cycle, and oxidative phosphorylation enzymes [26]. In this same species, LA supplementation levels from 1000 to 5000 mg Kg^−1^ of feed resulted in biphasic behavior of the expression of the enzymes hexokinase, phosphofructokinase, and pyruvate kinase, with the highest expression at intermediate LA concentrations. This behavior was also observed for isocitrate dehydrogenase expression, indicating an LA-induced hormetic-type effect [58]. Throughout the experiment, notable increases in lactate concentration were observed in organisms exposed to lactic acid. Under certain conditions and concentrations, LA may increase energy metabolism above respiratory capacity, which could be compensated for by activating metabolic pathways such as anaerobic glycolysis [59]. However, this should be explored in future studies because of the lack of previous data indicating that LA increases lactate concentrations, even when considering studies on other crustaceans [26,58]. Evidence for an increase in metabolic activity caused by LA was found in the ETS capacity results. During the experiment, this parameter increased according to the LA concentration, as shown by the PCA results (Figure 6b), where a highly positive correlation between ETS capacity and LA inclusion in the medium was observed. However, the mechanisms by which this occurs remain unclear. Nevertheless, there is evidence for the participation of the reduced nucleotides NADH and NADPH in LA reduction. Several proteins, including thioredoxin reductase, lipoamide dehydrogenase, and the E3 subunit of α-ketoglutarate dehydrogenase, can catalyze this reduction [60]. This process could imply a decrease in cytoplasmic and mitochondrial NAD(P)H/NAD(P)^+^ ratios. Mitochondrial ADP and AMP concentrations should increase under these conditions, favoring the consequent activation of proteins such as AMPK, which promotes the catabolic metabolism of organic macromolecules for the release of energy substrates, such as glucose or fatty acids, to restore ATP levels [25,53,61]. One effect observed after LA supplementation was increased sirtuin 1-SIRT1 expression [22]. This NAD^+^-dependent deacetylase can also increase the activity of transcription factors such as peroxisome proliferation-activated receptor gamma (PPARγ), which acts as an agonist in mitochondrial biogenesis, with potential consequences in terms of increasing ETS capacity [62].

This study did not show a consistent effect of LA on the antioxidant capacity, unlike the results observed in several other animal models. States of lower antioxidant capacity were observed at 6 and 18 h, whereas others had higher antioxidant capacity at 12 and 24 h, although at the latter time, this occurred only at the concentration of 10 µM LA (Figure 5). These different responses reflect the dual role of LA as a pro-oxidant and antioxidant, which explains the gradual decrease in the antioxidant capacity observed at 6 h as the concentration of LA increased. It should be considered that the increments in ETS promoted by adding LA to the medium could have influenced the antioxidant capacity results. Thus, these results can be strongly linked to the effects of LA as a promoter of energy metabolism as an increase in oxidative phosphorylation can cause increased ROS production [63], which, in principle, should be intercepted as a protective mechanism by antioxidant defenses [64]. Alpha-lipoic acid, as an agent that modulates the redox state of a cell by influencing the concentration of reduced nucleotides NAD(P)H, has the ability to meddle with energy production routes by altering the equilibrium of NAD^+^/NADH concentrations [25]. A relative surge in NAD^+^ concentrations can stimulate mitochondrial activity to replenish reduced nucleotides serving as energy transporters for the electron transport chain [65]. It is crucial to note that there is evidence of redox-type interactions between alpha-lipoic acid and insulin receptors, which in turn boost insulin signaling within cells [66].

The demand for endogenous antioxidants in situations of high ROS production can decrease the antioxidant capacity [67]. This could explain the metabolic increment associated with the energetic cost of antioxidant synthesis to cope with the ROS increase. This idea is based on evidence of the intimate relationship between AMPK activation and antioxidant gene expression, where the post-transcriptional changes performed by this protein on erythroid nuclear factor 2-related factor 2 (Nrf2) cause it to migrate to the nucleus, where it acts as a ligand on AREs to activate its expression [68]. LA induces metabolic changes (glucose and ETS output) in animals, which implies accelerated use of energy reserves that may interact with (or antagonize) normal physiological demands at this stage of life. In this situation, the organism could be forced to prioritize physiological functions differently than a more slowly developing organism and/or an organism with a constant nutrient intake. Further studies are needed to determine how LA acts in animals at an accelerated developmental stage with limited nutrient availability.

In the PCA analysis (Figure 6), owing to the high correlation of PC1 with time, it was possible to determine that this factor contributed the most to the variability observed within the data, where, in general, there was a decrease in nutrient concentration as the experiment progressed. The correlation between treatment with LA and PC2 provided evidence of the role of LA in data variability. Finally, it should be noted that the negative correlation between glucose and LA concentrations may indicate the influence of LA as a promoter of glucose catabolism (Figure 6a), possibly through positive regulation of mitochondrial energy activity. Additionally, at 24 h, a gradual decrease in ROS production was observed with the addition of LA, although the differences were only significant between the SCtrl and 10 µM treatment groups. LA did not promote antioxidant capacity, but paradoxically, it seemed to have had a sizeable effect on ROS production. This seems counterintuitive given its high ETS activity and low antioxidant capacity. However, considering that ROS production was lower than in SCtrl, the decrease in antioxidant capacity in LA-treated animals may have been the result of decreased ROS production as an effect of LA, which would be an adaptive response for the organism. Additionally, the observed increase in ETS activity may have influenced the decrease in the antioxidant capacity to contain ROS, a product of mitochondrial respiration.

To summarize the main findings observed in this study, we can mention that: (1) LA induced a higher aerobic metabolism, as indicated by the electron transport system (ETS) activity, pointing to its role as a metabolic regulator; (2) the in vivo ETS results can be associated mainly with the mitochondrial activity of artemia; (3) additionally, LA promoted glucose catabolism; (4) LA promoted a decrease in ROS production, as is consistent with its well-known antioxidant properties; (5) some combinations of LA concentrations (0.5 µM) and exposure time (18 h) induced higher protein levels and lowered TAN production.

## Data Availability

The data that support the findings of this study are openly available in Figshare at https://doi.org/10.6084/m9.figshare.23690880.v1.
